# Skewing information flow through pre- and postsynaptic plasticity in the mushroom bodies of *Drosophila*

**DOI:** 10.1101/lm.053919.124

**Published:** 2024-05

**Authors:** Carlotta Pribbenow, David Owald

**Affiliations:** 1 Institute of Neurophysiology, Charité–Universitätsmedizin Berlin, corporate member of Freie Universität Berlin and Humboldt-Universität zu Berlin, 10117 Berlin, Germany; 2 Einstein Center for Neurosciences Berlin, 10117 Berlin, Germany; 3 NeuroCure, Charité–Universitätsmedizin Berlin, corporate member of Freie Universität Berlin and Humboldt-Universität zu Berlin, 10117 Berlin, Germany

## Abstract

Animal brains need to store information to construct a representation of their environment. Knowledge of what happened in the past allows both vertebrates and invertebrates to predict future outcomes by recalling previous experience. Although invertebrate and vertebrate brains share common principles at the molecular, cellular, and circuit-architectural levels, there are also obvious differences as exemplified by the use of acetylcholine versus glutamate as the considered main excitatory neurotransmitters in the respective central nervous systems. Nonetheless, across central nervous systems, synaptic plasticity is thought to be a main substrate for memory storage. Therefore, how brain circuits and synaptic contacts change following learning is of fundamental interest for understanding brain computations tied to behavior in any animal. Recent progress has been made in understanding such plastic changes following olfactory associative learning in the mushroom bodies (MBs) of *Drosophila*. A current framework of memory-guided behavioral selection is based on the MB skew model, in which antagonistic synaptic pathways are selectively changed in strength. Here, we review insights into plasticity at dedicated *Drosophila* MB output pathways and update what is known about the plasticity of both pre- and postsynaptic compartments of *Drosophila* MB neurons.

Across insects, olfactory associative learning is dependent on an intact bilateral third-order neuropile called the mushroom bodies (MBs) (Menzel et al. 1974; Erber et al. 1980; Heisenberg et al. 1985; de Belle and Heisenberg 1994; Arican et al. 2023). Although bees, flies, and other insects display intricate behaviors and skills, several insights on MB function are derived from studying the model organism *Drosophila melanogaster* (vinegar fly; hereafter, *Drosophila* or fly). *Drosophila* can recall learned associative olfactory information for more than 24 h and is amenable to genetic manipulations. Indeed, an exceptional genetic toolbox permits the targeting and manipulation of single cells and networks. Cell-specific manipulations range from opto- and thermogenetic activation and inhibition to molecular interventions using RNA interference (for review, see Owald et al. 2015b). Moreover, the recent emergence of extended connectomes has largely facilitated predicting the functional logic of circuits (Eichler et al. 2017; Zheng et al. 2018; Li et al. 2020) based on a snapshot of highly resolved neuronal architecture.

Flies can learn to associate an odor with either reward (e.g., sugar) or punishment (e.g., electric shock) (Quinn et al. 1974; Tully and Quinn 1985; Schwaerzel et al. 2003; Krashes et al. 2007; Colomb et al. 2009). Recalling information about previous pairings leads to flies preferentially approaching or avoiding an odor (CS+, odor paired with reinforcer during training) over another (CS−, no pairing with reinforcer during training). This behavioral choice depends on both a CS+ as well as a CS− memory, which are formed in parallel and integrated subsequently (Jacob and Waddell 2020; Felsenberg 2021).

The *Drosophila* MBs, with their approximately 2000 intrinsic neurons (Kenyon cells, KCs) each, can be subdivided into roughly 15 compartments (Tanaka et al. 2008; Aso et al. 2014a; Takemura et al. 2017; Li et al. 2020). As KCs sparsely respond to odors, they can cover a large olfactory coding space (Turner et al. 2008; Honegger et al. 2011; Lin et al. 2014; Bielopolski et al. 2019; Bilz et al. 2020). KCs relay olfactory information and converge onto approximately 35 downstream MB output neurons (MBONs) (Tanaka et al. 2008; Aso et al. 2014a; Li et al. 2020). Individual KCs can compete in activating downstream MBONs allowing not only for coding odor identity or mixture profiles but also for coding changes in odor concentration (Vrontou et al. 2021). Notably, in *Drosophila* and other insects (Heisenberg 1998; Strausfeld et al. 1998), KCs can also respond to visual cues, and γd KCs of the *Drosophila* MBs are specifically involved in storing associative visual memories (Vogt et al. 2014, 2016). Combining associative visual with olfactory training leads to the binding of the two modalities resulting in engrams and strong memory performance upon retrieval (Thiagarajan et al. 2022; Okray et al. 2023). MBs also serve as an acute integration center (Groschner et al. 2018) for competing inputs like innately aversive CO_2_ and attractive food-associated odors (Lewis et al. 2015) and receive motor feedback signals (Zolin et al. 2021).

The MB output compartments are anatomically separated from each other by distinct innervation patterns of dopaminergic (DAergic) neurons (DANs). Axons of individual KCs pass through several of these compartments and form *en passant* synapses, allowing for compartment-specific synaptic changes (Tanaka et al. 2008; Aso et al. 2014a; Owald and Waddell 2015; Li et al. 2020). Learning-related plasticity itself predominantly takes place at the KC–MBON synapse (Séjourné et al. 2011; Pai et al. 2013; Plaçais et al. 2013; Bouzaiane et al. 2015; Cohn et al. 2015; Hige et al. 2015; Owald et al. 2015a; Perisse et al. 2016; Handler et al. 2019; Hancock et al. 2022): Odor-specific KC activation in temporal proximity to DA released from DANs (Tomchik and Davis 2009; Boto et al. 2014; Yamagata et al. 2015; Cervantes-Sandoval et al. 2017; Handler et al. 2019) leads to a modification of odor-evoked activity in MBONs. Depending on the training regime, KC–MBON synapses can either depress or potentiate (Séjourné et al. 2011; Cohn et al. 2015; Owald et al. 2015a; Perisse et al. 2016; Handler et al. 2019; Hancock et al. 2022; Pribbenow et al. 2022), resulting in a change of information flow the next time the animal encounters an odor. In parallel, axo-axonal KC synapses activated by an odor provide a lateral inhibition motif (Manoim et al. 2022) that allows for the maintenance of a sparse activity code along the KC terminals.

The type of DA receptor and the temporal patterning of olfactory input and DA release define the directionality of bidirectional modifications at KC–MBON synapses (Handler et al. 2019). Moreover, DANs release nitric oxide (NO) in addition to DA. NO counteracts DA-mediated plasticity and limits the time course of DA-induced plasticity traces (Aso et al. 2019). Glutamate released from glial cells also plays a role in regulating plasticity in the MBs via NMDA receptor (NMDAR) signaling (Miyashita et al. 2012, 2023). Importantly, glia-derived alanine provides the energy for KC plasticity during memory formation (de Tredern et al. 2021; Silva et al. 2022; Rabah et al. 2023).

How changes in synaptic strength across the MBs can guide behavior is formulated in the MB skew model (Owald and Waddell 2015). This framework posits that odor-evoked activity of antagonistic MB output pathways are changed following learning and are depressed or potentiated, depending on whether the MBONs belong to a class in which stimulation promotes approach or avoidance behavior (Aso et al. 2014b; Owald et al. 2015a). At the physiological level, this translates to approach KC–MBON connections getting strengthened following appetitive conditioning and weakened by aversive conditioning. On the contrary, odor-triggered activation of avoidance MBONs is enhanced following aversive conditioning and decreased following appetitive conditioning (Owald and Waddell 2015; Owald et al. 2015a). As single KCs feed parallel MBON pathways *en passant*, and synaptic strength onto different MBONs can be modulated selectively, later convergence of avoidance- and approach-promoting MBONs with differing degrees of input strength will result in a skew of information delivered to downstream targets (Owald and Waddell 2015).

MB-guided behavior is also subject to internal states and regulated by hunger and previous exposure to nutrients (Perisse et al. 2016; Pardo-Garcia et al. 2023). Importantly, the compartmentalized memory system enables state-dependent control of behavior: For instance, disinhibition motifs gating MB output pathways are regulated by hunger (Perisse et al. 2016; Sayin et al. 2019), whereas other MBON pathways regulate sleep (Sitaraman et al. 2015; Chouhan et al. 2021; French et al. 2021). Compartmentalization further allows for the writing of parallel memories with different half-lives (Bouzaiane et al. 2015; Aso and Rubin 2016). The way MBs integrate appetitive and aversive memories plays a decisive role for understanding the neural bases of addiction models to alcohol (Scaplen et al. 2020).

## Updating information

The MB skew model might also provide the basis for understanding how previously acquired information can be updated (Schwaerzel et al. 2002; Felsenberg et al. 2017, 2018; Davis 2023). Although it is evolutionarily important for animals to make predictions of an outcome in the future, it is likewise crucial to be able to update previous memories or to correct wrong predictions. Indeed, during memory extinction, parallel but opposing memories are written at the level of KC to MBON synapses. More precisely, to extinguish an appetitive memory, a new aversive memory is formed, and vice versa for aversive memory extinction. Importantly, the new opposing memory is stored in a separate neuronal pathway (Felsenberg et al. 2017, 2018). In accordance with the MB skew model, these two opposing memory pathways will be integrated downstream from the KC–MBON synapses. Thus, by writing an opposing memory the skew is balanced out again and the animal no longer displays a learned behavior. This remarkable example of plasticity demonstrates how adjusting opposing traces can lead to more complex computations. It is worth noting that the extinction of an aversive memory uses the same motif required not just for writing aversive memories but also for recalling appetitive memories in hungry flies (Perisse et al. 2016). Indeed, disinhibition signaling through MBON–MBON contacts is a prime example for MB cross-compartment communication (Perisse et al. 2016; Felsenberg et al. 2018). Cross-compartment signaling also mediates second-order conditioning in *Drosophila*, in which an odor is initially paired to a reinforcer. Subsequently, the odor information tied to a specific valence can itself serve as a reinforcer for additional odors (König et al. 2019; Yamada et al. 2023). The omission of a reinforcer can lead to reversal learning as well as reversal of MBON responses to odor cues (McCurdy et al. 2021).

The transient nature of DA-mediated memories allows for updating memories, but also forgetting (Berry et al. 2018; Sabandal et al. 2021). The scaffolding protein Scribble physically interacts with the Rho GTPase Rac1, the protein kinase Pak3, and the actin-modulating protein Cofilin to form a signalosome in KCs. Together these factors are required for active and interference-based forgetting. Notably, Scribble-based forgetting is regulated by DANs (Cervantes-Sandoval et al. 2016) and the scaffold Scribble is also required for NO-dependent memory (Aso et al. 2019). In specific DANs, the protein Sickie physically interacts with the active zone scaffold Bruchpilot (BRP) and regulates its abundance to regulate forgetting (Zhang et al. 2022). Interestingly, sleep counteracts dopamine-induced forgetting and therefore facilitates memory retention (Berry et al. 2015).

## Physiological plasticity

MBONs across several output compartments generally display calcium transients when a fly is exposed to an odor. In many cases, with some exceptions (Hige et al. 2015; Stahl et al. 2022), the peak or integrated amplitude change over time of such calcium transients is used as a proxy for synaptic efficacy throughout the MB literature. Indeed, several studies have shown that associative training (or associative training–like protocols) can reduce (and sometimes increase) odor-evoked calcium transients when the flies get reexposed to the CS+ (Séjourné et al. 2011; Pai et al. 2013; Plaçais et al. 2013; Cohn et al. 2015; Hige et al. 2015; Perisse et al. 2016; Handler et al. 2019; Hancock et al. 2022; Pribbenow et al. 2022). For example, both calcium responses to the CS+ in relation to the CS− and the response to the CS+ are decreased in MBON-γ1, pedc (MVP2 MBON) following training (Perisse et al. 2016; Hancock et al. 2022), which responds to decreased firing activity in electrophysiological experiments using optogenetic stimulation (Hige et al. 2015).

At least in some cases, odor-evoked activity in MBONs can also get modified bidirectionally (Cohn et al. 2015; Owald et al. 2015a; Handler et al. 2019), depending on the training protocol or timing of odor-evoked and DAergic signals. For example, CS+ induced calcium transients of MBON-β′2mp (M4 MBONs) decrease relative to CS− induced responses following appetitive training. In contrast, CS+ induced responses are increased (at least partially via disinhibition; Perisse et al. 2016) following aversive training (Owald et al. 2015a). In addition, artificial activation of DANs paired with odor-induced KC activation decreases or potentiates calcium responses in γ4 MBONs depending on the temporal coincidence of the two input signals (Cohn et al. 2015).

Not only memories following classical conditioning, but also other forms of learning can change odor-triggered calcium transients of selected MBONs. Depression of the α′3 KC–MBON synapse following repeated exposure of the animal to the same odor was shown to encode familiarity learning (Hattori et al. 2017) measured by changes in grooming activity in response to an unknown versus a familiar odor. The observed depression is manifest at the postsynaptic compartment, but not at the level of KC presynapses (Hattori et al. 2017; Pribbenow et al. 2022).

## Presynaptic plasticity

In vertebrate brains, learning-relevant synaptic plasticity has mainly been localized to glutamatergic connections (Korte and Schmitz 2016). In *Drosophila*, however, the identity of the fast excitatory neurotransmitter at KC output synapses remained unclear for a long time. Candidates identified included neuropeptides and sNPF was identified as a KC-derived neuromodulator shaping appetitive olfactory memory (Knapek et al. 2013; Barnstedt et al. 2016). Other candidates included glutamate and GABA (Johard et al. 2008; Sinakevitch et al. 2010; Gatto et al. 2014) based on immunoreactivity of KC subsets. However, the main fast neurotransmitter turned out to be acetylcholine (Barnstedt et al. 2016). How does this neurochemical difference—memory storage at glutamatergic versus cholinergic synapses—translate to potential commonalities or differences of plasticity mechanisms between vertebrates and invertebrates? Both the presynaptic neurotransmitter release machinery and active zone structure are largely conserved (Owald and Sigrist 2009; Südhof 2012; Walter et al. 2018) between vertebrates and invertebrates. Likewise, nicotinic acetylcholine receptors (nAChR) and glutamatergic AMPAR/NMDAR are evolutionarily conserved. However, nAChRs are cys-loop family receptors and therefore molecularly distinct from ionotropic glutamate receptors (excluding the insect glutamate-gated chloride channel) (Cully et al. 1996). Could such molecular differences of receptor types be reconciled by synaptic plasticity only taking place at the presynapse in invertebrates and pre- and postsynaptically in vertebrates, a notion previously proposed but challenged by others (Glanzman 2010)?

Indeed, changes at the level of presynaptic KC boutons following learning are well established in *Drosophila* (Ehmann et al. 2018). Experiments directly assaying changes in acetylcholine levels at KC terminals uncovered learning-induced changes in neurotransmitter released (Stahl et al. 2022). This is in line with observed changes of calcium transients in KCs following the pairing of DAergic signals and KC activation (Cohn et al. 2015; Handler et al. 2019). However, learning also was shown to induce a decorrelation of calcium transients across synaptic boutons of KC axons in the γ lobe compartments (Bilz et al. 2020). Therefore, evidence of presynaptic long-term changes at individual boutons, but also of relative changes between bouton profiles exist in the context of memory formation. Indeed, it was found that only strongly activated boutons would undergo long-term depression following learning and that the overall calcium transients changed differently across KC boutons (Bilz et al. 2020; Davidson et al. 2023). Thus, changes in presynaptic calcium transients appear more complex than a mere reduction or increase and need to be integrated with changes in cAMP levels that also have been observed following learning (Boto et al. 2014; Handler et al. 2019). However, in line with changes of MBON activity, presynaptic modifications seem to result in lasting changes of odor-evoked neurotransmitter release that can lead to either long-term depression or potentiation.

A wealth of evidence is available for molecular factors that are involved in memory formation at the presynaptic terminal. The Ca^2+^/CaM-responsive adenylyl cyclase Rutabaga (Rut-AC) and the antagonistic cAMP phosphodiesterase (PDE) Dunce are essential for memory formation (Dudai et al. 1976; Byers et al. 1981; Dudai 1983; Chen et al. 1986; Levin et al. 1992; Trannoy et al. 2011; Scheunemann et al. 2012; Walkinshaw et al. 2015). Although Rut-AC has been shown to increase cAMP in KCs following artificial training (Tomchik and Davis 2009), both Rut-AC and Dunce regulate synaptic size and vesicle release at the *Drosophila* neuromuscular junction (Kuromi and Kidokoro 2000; Renger et al. 2000; Ueda and Wu 2009). Of note, axonal localization of the kinase CaMKII in KCs is required for memory formation (Chen et al. 2022).

**Figure 1.**
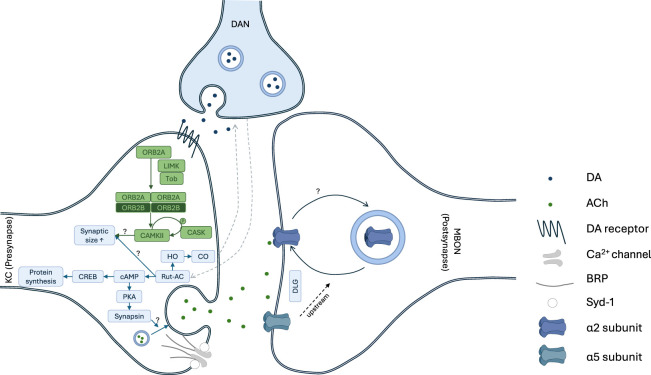
Overview of pre- and postsynaptic plasticity pathways at the KC–MBON synapse. Memory-relevant synaptic plasticity involves presynaptic KCs, postsynaptic MBONs and DANs. At the presynapse, the Rut-AC and Dunce regulate cAMP levels, which have been proposed to be involved in coincidence detection. The Rut-AC also activates the heme oxygenase which in turn leads to a CO production in KCs required for CO-dependent on-demand dopamine release from DANs (Ueno et al. 2017, 2020; Saitoe et al. 2022). Increase in cAMP levels, moreover, leads to PKA activation in KCs (Gervasi et al. 2010) following pairing of dopamine injection and KC depolarization (Tomchik and Davis 2009). One of the downstream targets of PKA might be Synapsin, which is required for certain memory phases (Knapek et al. 2010). At the *Drosophila* neuromuscular junction, Synapsin is required both for synaptic vesicle pool size and vesicle release and might have the same function in KCs (Akbergenova and Bykhovskaia 2010) following dopaminergic stimulation (Krüttner et al. 2015). ORB2 forms amyloid-like oligomers (Majumdar et al. 2012) in KCs. ORB2 further interacts with Tob to form oligomers. This oligmomerization is enhanced by the Lim kinase (White-Grindley et al. 2014). ORB2 regulates the synthesis of CAMKII (Krüttner et al. 2015), which autophosphorilates under CASK control (Malik and Hodge 2014). CAMKII regulates axonal growth at the NMJ and therefore potentially also in KCs (Nesler et al. 2016). Additionally, it is required for associative memories in KCs (Chen et al. 2022). Active zone proteins, including the calcium channel Cacophony, the scaffolds BRP, Syd-1, and Spinophilin, and the release factor Unc13 as well as the synaptic vesicle protein Synapsin, have been found to be required for different memory phases (Böhme et al. 2019; Turrel et al. 2022; Ramesh et al. 2023). On the postsynaptic side, the nAChR subunits α2 and α5 are required for appetitive learning. While the α5 subunit shows no sign of memory-related rearrangements, α2 subunit dynamics can be modified. Both the α5 subunit and Dlg, furthermore, seem to act upstream of α2 subunit-containing nAChRs (Pribbenow et al. 2022).

In recent years, a significant number of additional presynaptic proteins and cascades has been implicated in memory storage. An interesting example (for further examples, see Fig. 1) is ORB2 (Davis 2023), which forms amyloid-like oligomers (Majumdar et al. 2012) in KCs following DAergic stimulation (Krüttner et al. 2015). ORB2 is required for the formation and retrieval of memories (Li et al. 2016), whereas it is required in γ KCs for the formation of lasting courtship suppression memories (Keleman et al. 2007), olfactory appetitive memory (Li et al. 2016), and, in MBON-α3 (V3 MBONs), 24-h olfactory memories following spaced training (Pai et al. 2013). While the ORB2A isoform is required for memory acquisition, the ORB2B isoform is necessary during courtship suppression memory consolidation in KCs (Krüttner et al. 2015).

The high degree of evolutionary conservation of both the neurotransmitter release machinery and scaffolding proteins of the active zone make these plausible components of general plasticity mechanisms across phyla. Importantly, presynaptic active zone components and synaptic vesicle proteins, known to be involved in plasticity mechanisms at the *Drosophila* neuromuscular junction, including the scaffold BRP, the calcium channel Cacophony, the synaptic vesicle protein Synapsin, or the neurotransmitter release factor Unc13, are required for distinct phases of memory formation and consolidation at KC terminals (Knapek et al. 2010; Michels et al. 2011; Böhme et al. 2019; Turrel et al. 2022). Indeed, release factors (such as Unc13) and synaptic vesicle proteins are involved in immediate (and longer lasting) plasticity events (Böhme et al. 2019), indicating that these mediate a fast adaptation of functional properties.

On the contrary, scaffolding proteins, such as BRP, seem to be required post-encoding to stabilize memory traces (Turrel et al. 2022). Indeed, BRP is dispensable for immediate aversive memory formation; however, it is required for the expression of later memory stages. Extensive work has demonstrated that BRP, the ortholog to mammalian ELKS/CAST, with additional functional similarity to mammalian Bassoon, organizes clustering of presynaptic calcium channels (Cacophony) and tethering of synaptic vesicles at the *Drosophila* neuromuscular junction (Kittel et al. 2006; Fouquet et al. 2009; Hallermann et al. 2010; Matkovic et al. 2013; Ehmann et al. 2014; Mrestani et al. 2021; Ghelani et al. 2023). Thus, the requirement of BRP for learning-induced plasticity likely marks the requirement for structural changes at the active zone core. The post-encoding requirement of active zone material was further corroborated by the identification of proteins involved in the transport of active zone precursors for the expression of later memory stages (Turrel et al. 2022).

Moreover, the active zone scaffolds Syd-1, a seed factor for active zone plasticity at the *Drosophila* neuromuscular junction that interacts with the *trans*-synaptic scaffolds Neurexin and Neuroligin (Owald et al. 2010, 2012), and Spinophilin were shown to antagonistically regulate later memory phases (Turrel et al. 2022; Ramesh et al. 2023). Therefore, memory-related plasticity at KC active zones can likely be divided into at least two phases: an initial phase in which the neurotransmitter release machinery is involved directly and a later phase that involves active zone scaffolds and remodeling. How exactly molecular rearrangements translate to the observed changes in calcium transients and neurotransmitter release at KC terminals, however, needs to be determined.

In addition to presynaptic plasticity at the KC–MBON synapse, presynaptic plasticity at the projection neuron (PN)–KC synapse (the input to the KC dendrites) has been studied as well. When PNs are silenced an increased number and size of microtubules and an increased density of active zones have been reported (Kremer et al. 2010). Prolonged deprivation of synaptic transmission from PNs additionally led to an increased bouton size (Pech et al. 2015). Moreover, the number of presynaptic PN boutons is plastic and can adjust to the amount of pre- and postsynaptic cells (Elkahlah et al. 2020). Of note, PN–KC plasticity is at the basis for conditioning of the proboscis extension reflex in honeybees, whereas KC–MBON plasticity has also been demonstrated in honeybees and cockroaches (Szyszka et al. 2008; Groh and Rössler 2020; Arican et al. 2023). Moreover, plasticity mechanisms are not restricted to KC–MBON or KC–KC synapses in the MBs either. Indeed, KC–DAN communication shapes direct paths of communication through axo-axonal contacts or feedback or feedforward loops via MBONs (Ichinose et al. 2015; Cervantes-Sandoval et al. 2017; Otto et al. 2020; Villar et al. 2022). The binding of olfactory and visual information in the MBs is mediated via a bridging serotonergic neuron (Okray et al. 2023). Serotonin and octopamine both play decisive roles in MB plasticity (Burke et al. 2012; Huetteroth et al. 2015; Scheunemann et al. 2018).

## Postsynaptic plasticity of cholinergic synapses

Besides overwhelming evidence for presynaptic plasticity at the *Drosophila* KC–MBON synapse, does postsynaptic plasticity also play a role in learning in invertebrates? Initially speaking against postsynaptic involvement in memory formation were experiments showing that blocking neurotransmitter release (using thermogenetic acute intervention; please see Owald et al. 2015b for review of tools) from KCs during aversive conditioning left subsequent memory performance unaffected. One could argue that if the postsynapse would not see the neurotransmitter during learning, plasticity likely would take place presynaptically. However, transiently blocking KC transmission, especially during appetitive training, actually turned out to interfere with memory writing (Krashes et al. 2007; Ichinose et al. 2015; Yamazaki et al. 2018; Pribbenow et al. 2022). Although this is no evidence for a requirement of the postsynapse in forming memory traces, it means that the postsynapse “sees” and therefore could “react” to incoming signals (Pribbenow et al. 2022). However, several studies have suggested that protein synthesis is required in MBONs for long-term memory (Pai et al. 2013; Wu et al. 2017; Widmer et al. 2018), which also is in line with a potential postsynaptic role of memory storage. In addition, changes in postsynaptic (dendritic MBON) calcium transients were observed following artificial training paradigms in explant brains, where only the postsynapse (but not the presynapse) was activated by injecting acetylcholine, while concurrently activating dopaminergic neurons (Pribbenow et al. 2022).

It is well established at mammalian glutamatergic synapses that rearrangements of distinct receptor types at the postsynaptic compartment (postsynaptic density) are substrates for long-term potentiation and depression. The hierarchical interplay of NMDAR and AMPA receptors (AMPARs) triggers the incorporation or removal of AMPARs into/from the postsynaptic membrane. This results in a change of sensitivity of the postsynaptic compartment upon incoming activity (Citri and Malenka 2008; Korte and Schmitz 2016; Nicoll 2017). Receptors at the postsynaptic densities are regulated by scaffolding proteins (including PSD-95) (Buonarati et al. 2019). Could similar mechanisms play a role at cholinergic synapses during memory writing in *Drosophila* MBONs?

The *Drosophila* genome encodes seven nAChR α subunits and three β subunits (Hermans-Borgmeyer et al. 1986; Bossy et al. 1988; Baumann et al. 1990; Sawruk et al. 1990a,b; Lansdell and Millar 2000; Schulz et al. 2000; Grauso et al. 2002; Lansdell and Millar 2002). Their gene products can either form homomeric or heteromeric pentamers (Chamaon et al. 2000; Schulz et al. 2000; Chamaon et al. 2002; Lansdell et al. 2012; Ihara et al. 2020). The nAChR α subunits are nonuniformly distributed throughout the MB with an overall high number in the γ lobe and β′1 compartment (Pribbenow et al. 2022). Which receptor compositions are characteristic for individual MBON dendrites, however, remains unknown. On a physiological and behavioral level, α1, α4, α5, and α6 nAChR subunits are required both for odor-evoked calcium transients in MBON-γ5β′2a and MBON-β′2mp (M4/6) dendrites and for naive odor avoidance behavior (Barnstedt et al. 2016). Importantly, distinct α subunits are required for writing appetitive memories in M4/6 MBONs. Although the α5 subunit is involved in early memory formation (immediate memory), the α2 (and α1) and α5 subunits are necessary for appetitive memory performance at later stages. This indicates that α5 could act upstream of α2 in inducing memory-related plasticity, whereas α2 is required for the expression and consolidation of later stages of memories, somewhat analogous to the roles for NMDARs and AMPARs, respectively, in vertebrates. In line with this, α2 protein levels are dependent on α5 receptors at MBON dendrites. A further analogy of plasticity mechanisms is the involvement of Dlg (the conserved PSD-95 ortholog), in regulating α2 protein levels at MBON dendrites (Pribbenow et al. 2022), potentially by interacting with the receptors at the postsynapse. Besides PSD-95's involvement in memory-related plasticity at glutamatergic vertebrate synapses, Dlg is involved in glutamate receptor plasticity at the *Drosophila* neuromuscular junction (Chen and Featherstone 2005; Thomas et al. 2010) and, in addition, is required in other pathways for associative memory formation in *Drosophila* (Bertin et al. 2022). Of note, at the level of α′3 MBONs (MBON-α′3ap, MBON-α′3m), postsynaptic plasticity induced by familiarity learning also requires the α5 nAChR subunit upstream of the α2 subunit (Pribbenow et al. 2022).

In summary, evidence for postsynaptic nicotinic receptor plasticity involved in learning and memory is surfacing. Similar to a protracted sequence of molecules required for initial memory induction and subsequent expression at the presynapse, nicotinic receptor subunits are required for different plasticity phases. Despite profound differences in specific molecular building blocks between vertebrates and invertebrates, parallels in the general logic of how postsynapses rearrange (Nicoll 2017) can be found.

## Outlook

Growing evidence suggests that both pre- and postsynaptic plasticity mechanisms coexist for skewing MB output pathways by partially closing (depression) or opening (potentiation) exit gates. However, it remains largely unclear how the pre- and postsynaptic compartments communicate to adjust for changes on the other side. Diffusible messengers such as NO or CO (Ueno et al. 2017, 2020; Aso et al. 2019; Saitoe et al. 2022) along with *trans*-synaptic molecules could be prime candidates to mediate communication pathways.

Although it is widely accepted that in mammals both memory-relevant pre- and postsynaptic plasticity mechanisms exist (Korte and Schmitz 2016; Nicoll 2017; Fukaya et al. 2023), research in *Drosophila* in the past largely set the focus on presynaptic plasticity mechanisms. Nevertheless, increasing evidence for postsynaptic plasticity has surfaced (Pai et al. 2013; Wu et al. 2017; Widmer et al. 2018; Pribbenow et al. 2022).

Among other factors, expression of synaptic plasticity can largely be attributed to (1) rearrangement or exchange of postsynaptic receptors, (2) formation of new connections between pre- and postsynaptic cells, and (3) changes in the amount of transmitter released at the presynaptic active zone.

Evidence for the formation of new connections (Elkahlah et al. 2020), as well as changes in neurotransmitter release (Stahl et al. 2022), have been reported in the *Drosophila* MBs, either at the PN–KC or the KC–MBON synapse, respectively. Likewise, rearrangement of nicotinic receptor subunits has been observed (Pribbenow et al. 2022) as all the above criteria would be met. At glutamatergic hippocampal synapses, NMDAR and AMPAR interplay is crucial for long-term synaptic changes (Nicoll 2017). At MBON dendrites, α5 subunit–positive nAChRs function upstream of α2 subunits. How (or not) the detailed interaction of the receptor subtypes in the MBs functionally compares to mechanisms found for mammalian/glutamatergic synapses remains to be determined. Importantly, glutamatergic signaling—for instance, via glutamatergic MBONs or via NMDAR—does play a role in the MBs, however, likely up- or downstream from memory storage at KC–MBON synapses. Further mechanistic insight is also especially relevant for differentiating pathways for long-term synaptic depression versus potentiation for postsynaptic plasticity mechanisms. Likewise, potential changes in receptor makeup or dynamics during forgetting will be of interest.

Sensory information enters the MBs and can be routed through several MB exit gates. Synaptic plasticity allows to prioritize some pathways over others. Dependent on which pathways get modified, the skew of information flow influences an animal's behavioral choice. An understanding of the downstream targets of MBONs, as well as feedback loops back to the MBs, will allow further insight into how signals leaving the MBs are computed. Some relevant pathways have been uncovered so far: For example, UpWind neurons were identified to integrate inhibitory and excitatory inputs from MBONs (Aso et al. 2023) to steer behavior. Moreover, connections to the central complex, which is involved in several processes, including sleep regulation (Raccuglia et al. 2019) and navigation (Hulse et al. 2021), are relevant in the context of memory consolidation, hunger signaling, and sleep (Chouhan et al. 2021; French et al. 2021; Lei et al. 2022; Matheson et al. 2022).

Although we here focus on MB pathways involved in memory writing, it is conceivable that olfactory information also gets integrated across other brain areas—for instance, the lateral horn. Likewise, as mentioned, the MBs are not specific for olfactory information. Further understanding how modality representation gets integrated across the brain following learning, potentially through circuit rearrangements, will be of considerable interest.
